# The use of Raman spectroscopy to differentiate between different prostatic adenocarcinoma cell lines

**DOI:** 10.1038/sj.bjc.6602638

**Published:** 2005-05-31

**Authors:** P Crow, B Barrass, C Kendall, M Hart-Prieto, M Wright, R Persad, N Stone

**Affiliations:** 1 Biophotonics Research Group, Pullman Court, Gloucestershire Royal Hospital, Great Western Road, Gloucester GL1 3NN, UK; 2 Department of Urology, Bristol Royal Infirmary, Marlborough Street, Bristol BS2 8HW, UK

**Keywords:** Raman spectroscopy, prostate, cancer, cell culture

## Abstract

Raman spectroscopy (RS) is an optical technique that provides an objective method of pathological diagnosis based on the molecular composition of tissue. Studies have shown that the technique can accurately identify and grade prostatic adenocarcinoma (CaP) *in vitro*. This study aimed to determine whether RS was able to differentiate between CaP cell lines of varying degrees of biological aggressiveness. Raman spectra were measured from two well-differentiated, androgen-sensitive cell lines (LNCaP and PCa 2b) and two poorly differentiated, androgen-insensitive cell lines (DU145 and PC 3). Principal component analysis was used to study the molecular differences that exist between cell lines and, in conjunction with linear discriminant analysis, was applied to 200 spectra to construct a diagnostic algorithm capable of differentiating between the different cell lines. The algorithm was able to identify the cell line of each individual cell with an overall sensitivity of 98% and a specificity of 99%. The results further demonstrate the ability of RS to differentiate between CaP samples of varying biological aggressiveness. RS shows promise for application in the diagnosis and grading of CaP in clinical practise as well as providing molecular information on CaP samples in a research setting.

Prostate cancer is the most common cancer diagnosed in males in the UK (Quinn *et al*, 2001). The gold standard technique for diagnosing and characterising prostate cancer is currently histological examination of prostate biopsy cores. The subjective nature of this technique means that it is associated with considerable inter- and intraobserver variation in reporting (Ozdamar *et al*, 1996; Allsbrook *et al*, 2001). As the therapeutic options available to treat prostate cancer increase, so does the importance of accurate characterization of the tumour. For this reason, the development of new technologies capable of providing accurate and objective methods to assess prostate cancer is desirable.

Raman spectroscopy (RS) is an optical technique that utilises molecular-specific, inelastic scattering of light photons to interrogate biological material (Stone *et al*, 2002; Crow *et al*, 2003a). When material is illuminated with laser light, a small fraction of the photons are inelastically scattered by the intramolecular bonds present. When this occurs, the photon donates energy to or receives energy from the molecule, producing a change in the molecule's vibrational state. When it subsequently exits the material, the light photon has an altered energy level and, therefore, a different wavelength compared to the original laser light. This change in the photon's energy is known as the ‘Raman shift’ and is measured in wavenumbers.

Photons interacting with different biochemical bonds undergo specific Raman shifts, which considered together form the ‘Raman spectrum’. The Raman spectrum is a plot of intensity against Raman shift (Figure 1). The Raman spectrum is a direct function of the molecular composition of the material studied. When applied to biological tissue, the technique holds the potential to differentiate between different pathologies based on the differences in their biochemical makeup.

A study of prostatic core biopsies has shown that RS can identify and grade prostatic adenocarcinoma (CaP) *in vitro* (Crow *et al*, 2003b), although the heterogeneity of prostatic pathology makes prostatic tissue difficult to study. Cultured human prostatic cell lines are the product of a monoclonal proliferation of immortalised CaP cells. All cells within each cell line are theoretically identical, making them easier to study than the heterogeneous prostatic tissue. The aim of this study was to determine whether RS is able to differentiate between cell lines representing CaPs of varying biological aggressiveness. The study was undertaken with a view to provide complimentary evidence to support the findings of the prostate biopsy studies with a view to develop the technique for application in clinical practise.

## MATERIALS AND METHODS

### Cell culture and preparation

Human prostate cancer cell lines (LNCaP, PC 3, DU145 and PCA 2b) were obtained from the European collection of Animal Cell Cultures Catalogue (ECACC) Porton Down, UK and grown in a humidified 5% CO_2_ atmosphere at 37°C according to the protocols suggested by the ECACC. DU145, LNCaP and PC 3 cells were maintained in RPMI 1640 tissue culture media (BioWhittaker™) with L-glutamine supplemented with 10% foetal calf serum (FCS), penicillin (5000 IU ml^−1^), streptomycin (5 mg ml^−1^) and L-glutamine (5 mg ml^−1^) (growth media). MDApca2b cells were maintained in RPMI 1640 tissue culture media without L-glutamine and without Phenol Red (Gibco, Paisley, UK) supplemented with cholera toxin (62.5 *μ*l), EGF (10 ng ml^−1^), 0.05 mM phosphoethanolamine (25 *μ*l) (100 pg ml^−1^ hydrocortisone) 250 *μ*l, 45 nM selenious acid (1 *μ*l), 0.005 mg ml^−1^ insulin (22 *μ*l), penicillin (5000 IU ml^−1^), streptomycin (5 mg ml^−1^) and 20% foetal bovine serum (100 ml) (growth media). The cells were transported in low serum RPMI 1640 tissue culture media (BioWhittaker™) supplemented with 0.1% FCS, penicillin (5000 IU ml^−1^), streptomycin (5 mg ml^−1^) and L-glutamine (5 mg ml^−1^) (serum-free media). Tissue culture plastics were obtained from Greiner Labortechnic Ltd (Stonehouse, UK).

A limited number of prostate cancer cell lines are available and the above four cell lines were selected as they can be considered to present various stages in the development of androgen-independent prostate cancer and of biological aggression. LNCaP (Horoszewicz *et al*, 1983) and MDApca2b (Navone *et al*, 1997) cells can be considered relatively well-differentiated cells, which retain androgen sensitivity, whereas DU145 (Mickey *et al*, 1980) and PC 3 (Kaighn *et al*, 1979) cells are more poorly differentiated cells, which no longer posses androgen sensitivity and show more aggressive phenotypic features such as increased migration and invasiveness (Lang *et al*, 2000; Fisher *et al*, 2002).

To be suitable for analysis by the Raman system, the cells required transfer to a calcium fluoride slide. Cells were seeded in a T-75 tissue culture flask at a density of 1 × 10^6^ cells ml^−1^ and grown to 80% confluence in growth media as described above. The growth media were then aspirated and replaced with 10 ml serum-free media and the cells were incubated for a further 24 h. The cells were scrapped free from their culture dish using a cell scraper (GrienerBio-one), transferred to a 20 ml universal tube and centrifuged at 2000 r.p.m. for 10 min at room temperature. Trypsin was avoided to minimise contamination of the samples. The supernatant was aspirated and the cells were resuspended in phosphate-buffered saline (PBS) (Oxoid BR14) to wash the remaining media off the cells. The cells were then centrifuged for 10 min at 2000 r.p.m. for a second time. The supernatant was then aspirated and the cells put through a cytospin centrifuge cycle at 1250 r.p.m. for 5 min, placing the cells directly onto a calcium fluoride slide. The slide was kept in a moist environment and taken back to the spectroscopy lab, so that spectra could be measured from the cells, within 30 min of removal from the culture medium.

### Raman spectroscopy

**Figure 2:**
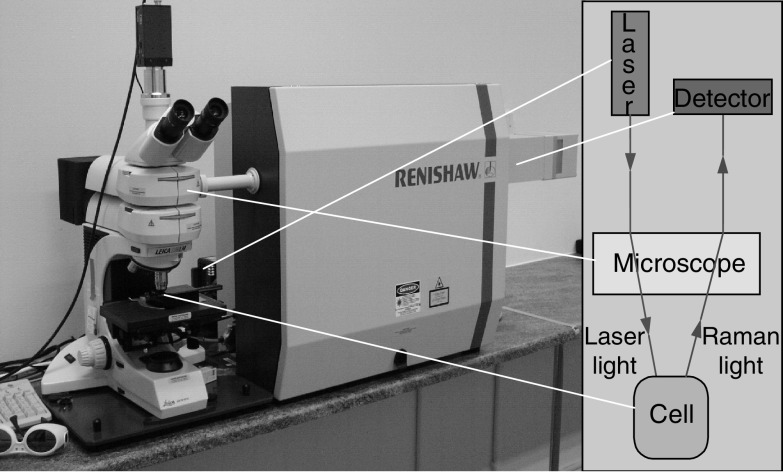
Renishaw 1000 Raman system.

The Raman system (Figure 2) comprises a 300 mW laser (Renishaw) that supplies near-infrared excitation light at 830 nm, which is focused onto the sample via a microscope objective (Leica NPLAN × 50 objective). The same objective collects the scattered light from the sample and directs it to the spectrometer. The spectrometer processes this scattered light, by rejecting the unwanted portion and separating the remainder into its constituent wavelengths. The Raman spectrum is recorded on a deep depletion charge-coupled device detector (Renishaw RenCam). The system has been customised in order to produce the optimal set-up for recording Raman spectra from biological tissue *in vitro* (Stone, 2001). The recorded Raman spectrum is digitalised and displayed on a personal computer using GRAMS/32 software (Galactic Industries). The system is managed using Renishaw WiRE software (Windows based Raman Environment Version 1.3.15), which allows the experimental parameters to be set.

In all, 50 Raman spectra were recorded from DU 145 cells and LNCaP cells, with 49 and 51 spectra recorded from MDApca2b and PC 3 cells, respectively. Each spectrum was acquired over a period of 20 s.

### Construction and testing of the diagnostic algorithm

The Raman spectra were analysed with the aim of determining whether there were consistent differences between the molecular composition of the different cell lines and whether these differences would allow for construction of a diagnostic algorithm capable of identifying the cell line of an individual cell from its Raman spectrum. The algorithms were created using principal component fed (PCA/LDA) as described below. This spectral analysis was carried out using tailor-made routines run in the MATLAB® environment and the Eigenvector® PLS-toolbox.

Principal component analysis was used to compress the information held by the spectra (Jackson, 1991) and involved calculating 25 principal components (also known as loads) that describe the greatest variance of the spectral data from its mean. Each spectrum can be reconstructed by adding the products of the loads and the scores for each spectrum. The scores are effectively the amount of each component found in the spectrum. Therefore, so long as the loads are known, the spectra in the data set can be described by the 25 scores. The first three principal components were visually analysed to determine whether there were differences in the concentration of specific species of molecules present in each cell line. The degree of separation achieved between the different cell lines was initially determined using PCA alone. Subsequently, LDA was also employed to accentuate the separation between cell lines (Ramanujam *et al*, 1996; Otto, 1999) in order to increase the diagnostic accuracy of the algorithm. This was achieved by applying three linear discriminant functions (LDFs) to each of the spectra.

The accuracy of the algorithm, in correctly identifying a cell's identity from its spectrum, was determined using ‘leave one spectrum out’ crossvalidation. This involved removing one spectrum from the data set and constructing a diagnostic algorithm from the remainder of the spectra. The algorithm then predicted the cell line of ‘left out’ spectrum and stored the result. This process was repeated, with each spectrum left out in turn, until each of the spectra had its cell line predicted. The overall predictive accuracy of the algorithm was calculated and expressed in terms of sensitivity and specificity for each cell line.

## RESULTS

**Figure 3:**
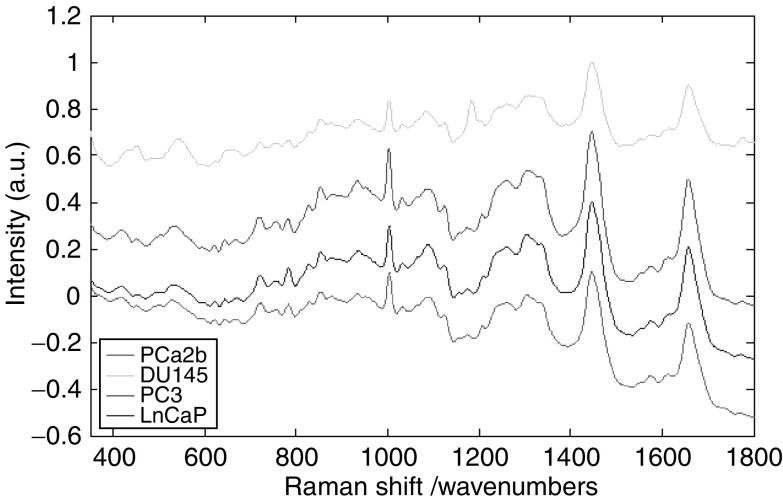
Mean Raman spectra measured from each of the cell lines.

**Figure 4:**
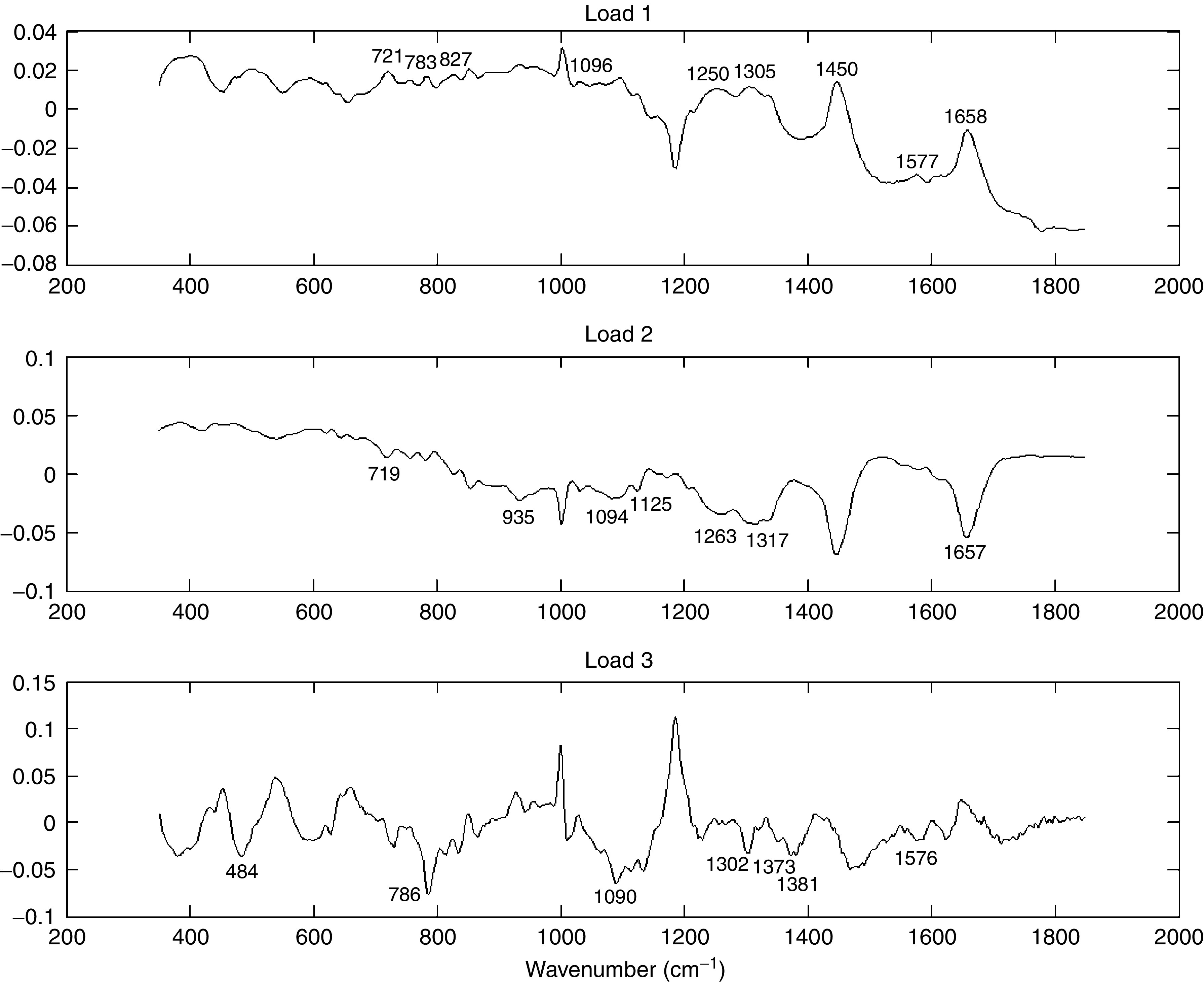
Loads of the first three principal components. These describe the greatest variance in the spectra.

Using the methods outlined above, good quality Raman spectra were measured from each of the cell lines studied. Figure 3 shows the mean Raman spectra measured from each of the four cell lines. In the interest of clarity, the spectra have been equally spaced with respect to the intensity axis utilising the 1450 peak as a reference point. Although the spectra appear similar in shape, subtle morphological differences exist. These differences were exploited by calculating the principal components of the spectral data set. The first three principal components, that is, those that describe the greatest variance in the spectral data set, are shown in Figure 4. Visual analysis of these principal components allows identification of molecular species from their Raman peaks. The principal components have peaks and troughs that can be identified from the literature to provide an understanding of the origins of the statistical variations. It is the concentrations of these molecular species that vary most significantly between the four cell lines.

Principal component 1 (PC1) represents increased concentrations of nuclear acids (721, 783, 1305, 1450 and 1577 cm^−1^), DNA backbone (O–P–O) (827 and 1096 cm^−1^) and unordered proteins (1250 and 1658 cm^−1^). Principal component 2 (PC2) represents decreased concentrations of *α*-helix proteins (935, 1263 and 1657 cm^−1^) and phospholipids (719, 1094, 1125 and 1317 cm^−1^). Principal component 3 (PC3) represents decreased concentrations of lipids (1090, 1302 and 1373 cm^−1^), glycogen (484 cm^−1^) and nucleic acids (786, 1381 and 1576 cm^−1^).

Cell lines DU145 and PC 3 are androgen insensitive and can be discriminated when the scores for PC3 are high and the scores for PC2 less than or equal to zero. This is in contrast to the androgen-sensitive cell lines of MDApca2b and LnCaP, which can be discriminated with PC3 scores of zero or less and PC2 scores of zero or greater.

**Figure 5:**
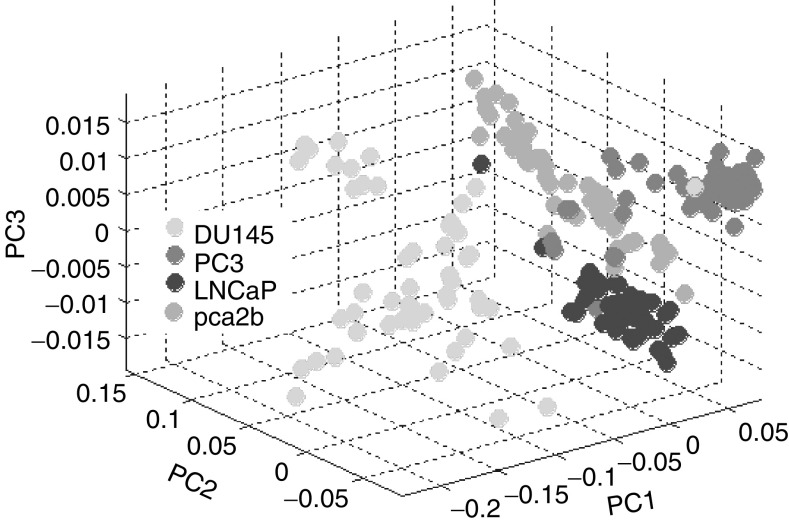
A three-dimensional scatter plot of the scores of PCs 1, 2 and 3. This demonstrates the natural clustering of the four cell lines spectra, without any modification of the algorithm to maximise group separation.

A good separation between the four cell lines can be achieved by utilising just the first three PCs. A three-dimensional scatter plot of the principal components scores (the amount of each spectral component found in each spectrum measured) is shown in Figure 5. This significantly strengthens the view that there is an inherent molecular difference between the cells, by providing a display of the natural separation of the data without supervised statistical manipulation. Cell line DU145 appears to have a distinctly lower level of nuclear material and unordered proteins when compared to the other cell lines, as represented by PC1.

**Figure 6:**
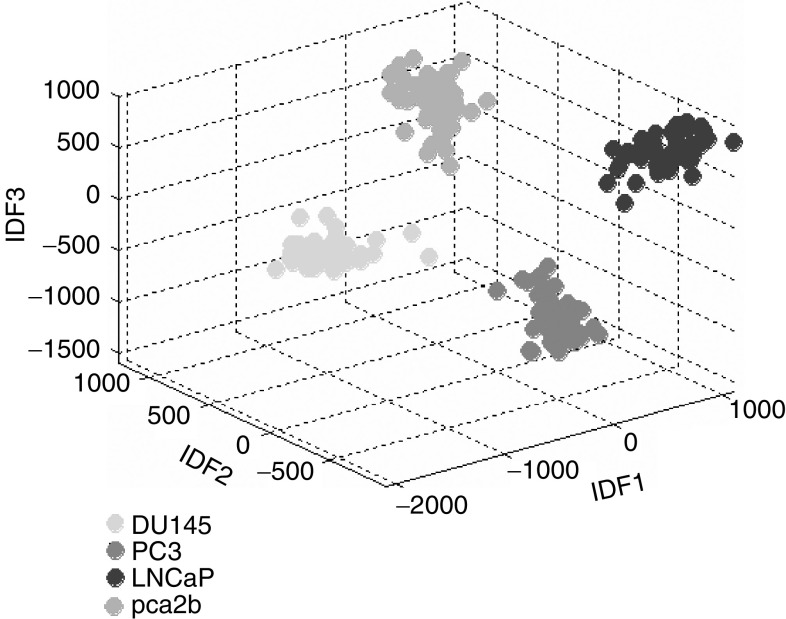
A three-dimensional scatter plot of the scores of LDFS 1, 2 and 3 demonstrating the clustering of the four cell lines achieved by the PCA/LDA algorithm.

Figure 6 illustrates the degree of separation that the PCA/LDA algorithm achieved between the spectra of each cell line. The position of each spectrum is plotted in three-dimensional space relative to each of its three LDFs. It can be seen that there is excellent clustering of spectra within each cell line group, with each of the four cell line groups occupying a distinct region of the three-dimensional plot space.

**Figure 7:**
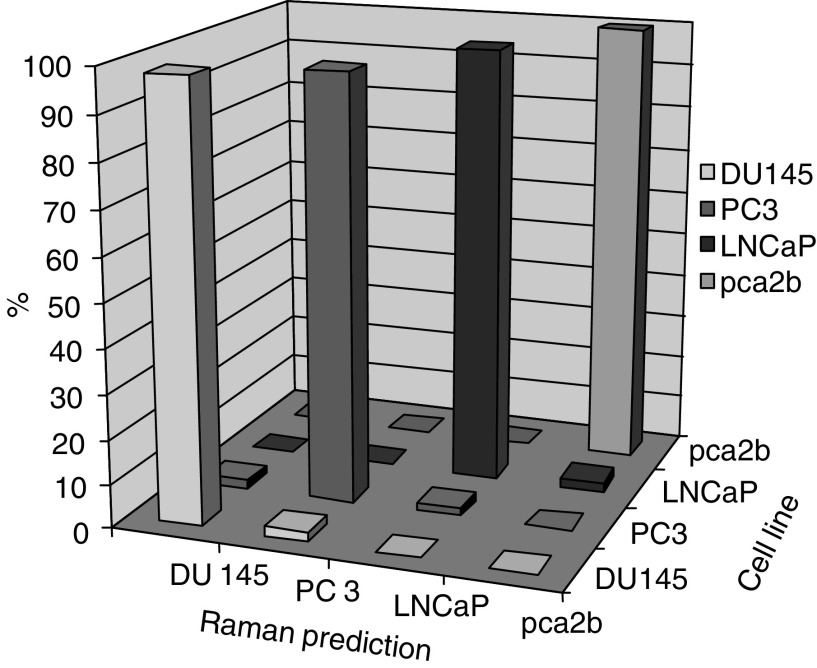
A bar chart demonstrating the prediction power of the diagnostic algorithm.

Table 1 gives the crossvalidated results achieved by the algorithm. The rows of the table show the numbers of spectra measured for each cell line. The columns of the table show the number of spectra that the algorithm has predicted as belonging to each cell line. By looking across each row of the table, the number of correctly predicted spectra for each cell line, shown in bold, can be seen. The remaining values within each row represent misclassifications by the algorithm. Figure 7 illustrates these data in bar chart form. The *X*-axis represents true cell line and the *Z*-axis represents Raman predicted cell line, with the *Y*-axis displaying the percentage of spectra predicted into each cell line. The diagonal row of large bars shows correct predictions by the algorithm, with all other bars representing misclassifications.

Table 2 shows how these results translate into sensitivities and specificities for the prediction of each of the four cell lines.

## DISCUSSION

The results of the PCA taken alone begin to give insight as to the types of molecules that vary in concentration between CaP cell lines of differing biological aggressiveness. These variations are complex, making it difficult to form a complete picture as to their nature; however, the major variations are documented in the results. In a previous Raman study, CaP was found to have a reduced concentration of glycogen and increased concentration of nucleic acids compared to benign prostatic hyperplasia (Crow *et al*, 2003b). The results of the current study suggest that lower glycogen concentrations are found in poorly differentiated cell lines (DU145 and PC3) compared to the better differentiated cell lines (MDApca2b and LnCaP). The picture with nucleic acids is less clear with the androgen-independent cell lines, if anything, showing lower concentrations of nucleic acids than androgen-dependent cell lines. Interpretation of the spectra and principal components remains imprecise and qualitative in nature; however, work is ongoing to devise an accurate method by which to retrieve quantitative molecular information.

The PCA/LDA algorithm achieved near perfect identification of each cell line, with sensitivities ranging from 96 to 100% and specificities all 99% or higher. These results confirm that RS is able to differentiate between cells derived from CaPs of different biological aggressiveness.

These findings suggest that RS may have potential application as a research tool. Within the field of cell culture, for example, when cells undergo apoptosis, the DNA content of the cell is reduced and flow cytometry is currently used to quantify apoptosis by measuring the DNA content of cells (Darzynkiewicz *et al*, 2001). As RS exploits biochemical differences between cells, an algorithm could be generated to identify apoptotic cells based on the change in their Raman spectrum as the DNA content diminishes.

This study also has implications for clinical practise. The results reinforce previous studies, which have suggested that RS can be used to grade CaP (Crow *et al*, 2003b). Given the high degree of accuracy and objectivity achieved, these findings strongly support the development of RS as a tool capable of assisting pathologists in accurately assessing prostatic tissue. Prior to the widespread use of core biopsy, many CaPs were diagnosed using prostatic fine-needle aspiration (FNA). Core biopsy has superseded FNA, largely because histological examination of prostatic core biopsies is able to provide more accurate prognostic information than cytological examination of prostatic FNA samples (Schmidt, 1992). The results achieved by this study demonstrate that RS may have utility analysing prostate FNA samples with the potential to derive prognostic information.

Whether applied to analyse prostate biopsies or FNA samples, the high speed, accuracy and objectivity of RS means that it shows promise in improving the clinical diagnosis of CaP.

## Figures and Tables

**Figure 1:**
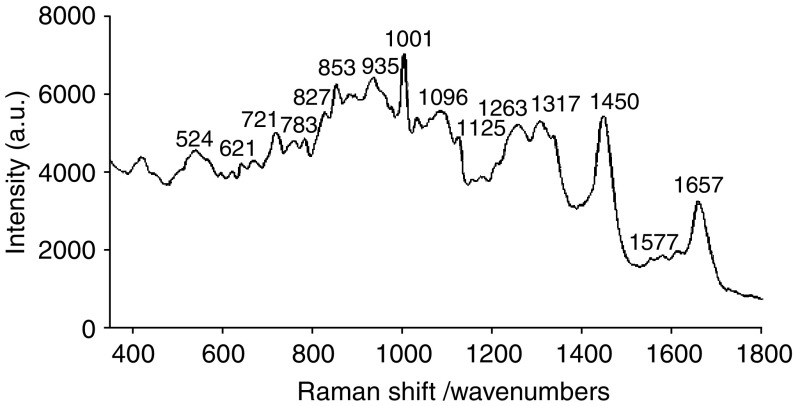
A Raman spectrum from a prostate cell with wavenumbers labelled for major peaks.

**Table 1:** Crossvalidated results achieved by the diagnostic algorithm

	**Raman prediction**			
	**DU145**	**PC3**	**LNCaP**	**pca2b**
*Confirmed histology*				
DU145	**49**	1	0	0
PC3	1	**49**	1	0
LNCaP	0	0	**49**	1
pca2b	0	0	0	**49**

**Table 2:** Sensitivities and specificities achieved by the diagnostic algorithm

	**DU145 (%)**	**PC3 (%)**	**LNCaP (%)**	**pca2b (%)**
Sensitivity	98	96	98	100
Specificity	100	99	99	99

## References

[bib1] Allsbrook Jr WC, Mangold KA, Johnson MH, Lane RB, Lane CG, Epstein JI (2001) Interobserver reproducibility of Gleason grading of prostatic carcinoma: general pathologist. Hum Pathol 32(1): 81–88 11172299 10.1053/hupa.2001.21135

[bib2] Crow P, Stone N, Kendall CA, Persad RA, Wright MPJ (2003a) Optical diagnostics in Urology: current applications and future prospects. Br J Urol Int 92: 400–407 10.1046/j.1464-410x.2003.04368.x 12930429

[bib3] Crow P, Stone N, Kendall CA, Uff JS, Farmer JAM, Barr H, Wright MPJ (2003b) The use of Raman spectroscopy to identify and grade prostatic adenocarcinoma *in vitro*. Br J Cancer 89(1): 106–108 12838309 10.1038/sj.bjc.6601059 PMC2394218

[bib4] Darzynkiewicz Z, Bedner E, Smolewski P (2001) Flow cytometry in analysis of cell cycle and apoptosis. Semin Hematol 38(2): 179–193 11309699 10.1016/s0037-1963(01)90051-4

[bib5] Fisher JL, Schmitt JF, Howard ML, Mackie PS, Choong PF, Risbridger GP (2002) An *in vivo* model of prostate carcinoma growth and invasion in bone. Cell Tissue Res 307(3): 337–345 11904770 10.1007/s00441-001-0503-x

[bib6] Horoszewicz JS, Leong SS, Kawinski E, Karr JP, Rosenthal H, Chu TM, Mirand EA, Murphy GP (1983) LNCaP model of human prostatic carcinoma. Cancer Res 43(4): 1809–1818 6831420

[bib7] Jackson JE (1991) A User's Guide to Principal Components. New York: Wiley

[bib8] Kaighn ME, Narayan KS, Ohnuki Y, Lechner JF, Jones LW (1979) Establishment and characterization of a human prostatic carcinoma cell line (PC 3). Invest Urol 17(1): 16–23 447482

[bib9] Lang SH, Stower M, Maitland NJ (2000) *In vitro* modelling of epithelial and stromal interactions in non-malignant and malignant prostates. Br J Cancer 82(4): 990–997 10732776 10.1054/bjoc.1999.1029 PMC2374381

[bib10] Mickey DD, Stone KR, Wunderli H, Mickey GH, Paulson DF (1980) Characterization of a human prostate adenocarcinoma cell line (DU145) as a monolayer culture and as a solid tumor in athymic mice. Prog Clin Biol Res 37: 67–84 7384095

[bib11] Navone NM, Olive M, Ozen M, Davis R, Troncoso P, Tu SM, Johnston D, Pollack A, Pathak S, von Eschenbach AC, Logothetis CJ (1997) Establishment of two human prostate cancer cell lines derived from a single bone metastasis. Clin Cancer Res 3(12): 2493–2500 9815652

[bib12] Otto M (1999) Chemometrics: Statistics and Computer Application in Analytical Chemistry. New York: Wiley-VCH

[bib13] Ozdamar SO, Sarikaya S, Yildiz L, Atilla MK, Kandemir B, Yildiz S (1996) Intraobserver and interobserver reproducibility of WHO and Gleason histologic grading systems in prostatic adenocarcinomas. Int Urol Nephrol 28(1): 73–77 8738623 10.1007/BF02550141

[bib14] Quinn M, Babb P, Brock A (2001) Cancer trends in England and Wales 1950–1999. Studies on medical and population subjects: Chapter 3. Natl Stat 66: 28–33

[bib15] Ramanujam N, Mitchell MF, Mahadevan A, Thomsen S, Malpica A, Wright T, Atkinson N, Richards-Kortum R (1996) Development of a multivariate statistical algorithm to analyze human cervical tissue fluorescence spectra acquired *in vivo*. Lasers Surg Med 19: 46–62 8836996 10.1002/(SICI)1096-9101(1996)19:1<46::AID-LSM7>3.0.CO;2-Q

[bib16] Schmidt JD (1992) Clinical diagnosis of prostate cancer. Cancer 70(1 Suppl): 221–224 1376191 10.1002/1097-0142(19920701)70:1+<221::aid-cncr2820701308>3.0.co;2-9

[bib17] Stone N (2001) Raman spectroscopy of biological tissue for application in optical diagnosis of malignancy. PhD Thesis, Cranfield University

[bib18] Stone N, Kendall C, Shepherd N, Crow P, Barr H (2002) Near-infrared Raman spectroscopy for the classification of epithelial pre-cancers and cancers. J Raman Spectrosc 33(7): 564–573

